# Ruthenium Polypyridyl Complex Inhibits Growth and Metastasis of Breast Cancer Cells by Suppressing FAK signaling with Enhancement of TRAIL-induced Apoptosis

**DOI:** 10.1038/srep09157

**Published:** 2015-03-17

**Authors:** Wenqiang Cao, Wenjie Zheng, Tianfeng Chen

**Affiliations:** 1 Department of Chemistry, Jinan University, Guangzhou 510632, China

## Abstract

Ruthenium-based complexes have emerged as promising antitumor and antimetastatic agents during the past decades. However, the limited understanding of the antimetastatic mechanisms of these agents is a roadblock to their clinical application. Herein, we reported that, RuPOP, a ruthenium polypyridyl complex with potent antitumor activity, was able to effectively inhibit growth and metastasis of MDA-MB-231 cells and synergistically enhance TRAIL-induced apoptosis. The selective intracellular uptake and cytotoxic effect of RuPOP was found associated with transferring receptor (TfR)-mediated endocytosis. Further investigation on intracellular mechanisms reveled that RuPOP notably suppressed FAK-mediated ERK and Akt activation. Pretreatment of cells with ERK inhibitor (U0126) and PI3K inhibitor (LY294002) significantly potentiated the inhibitory effect of RuPOP on cell growth, migration and invasion. Moreover, the alternation in the expression levels of metastatic regulatory proteins, including uPA, MMP-2/-9, and inhibition of VEGF secretion were also observed after RuPOP treatment. These results demonstrate the inhibitory effect of RuPOP on the growth and metastasis of cancer cells and the enhancement of TRAIL-induced apoptosis though suppression of FAK-mediated signaling. Furthermore, RuPOP exhibits the potential to be developed as a metal-based antimetastatic agent and chemosensitizer of TRAIL for the treatment of human metastatic cancers.

Breast carcinoma is the most common cause of malignancy, with various subtypes exhibiting a number of biological behaviors and molecular characteristics^1^. As a subtype of breast cancer, Triple-negative breast cancer (TNBC) is negative for expression of progesterone receptor (PR), estrogen receptor (ER), and human epidermal growth factor receptor 2 (HER-2), and accounts for 10% to 20% of all breast cancer cases^2^. TNBC is highly aggressive, correlated with rapid relapse after medical treatment, lower survival rate and increased metastasis to the brain and distant organs^3^. The limited efficacy of current systemic treatment for TNBC promotes the development of novel agents with notable antimetastatic potential that could modulate the tumor metastasis signaling pathways and the related metastatic regulatory proteins.

Tumor metastasis is a multistep biological process that allows cancer cells to get away from the primary tumor, survive in the circulation, located in distant sites and grow^4^. Successful metastasis is relay on the balance of both the metastasis activators and inhibitors in the whole process. As a group of zinc-dependent endopeptidases, matrix metalloproteinases (MMPs), was found to have potential in extracellular matrix (ECM) degradation and thus correlated with the late stages of tumor invasion and metastasis^5^. The activation of MMPs are directly regulated by plasmin, which is activated by the serine protease urokinase-type plasminogen activator (uPA)/uPA receptor (uPAR) system^6^. Furthermore, the expression levels of uPA/uPAR and MMPs are positively regulated by a variety of upstream signaling pathways, such as Ras/MEK/ERK and PI3K/Akt pathway. In addition, as a widely expressed tyrosine kinase, focal adhesion kinase (FAK) plays an critical role in the regulation of various biological processes, including cell growth, migration and invasion^7^. Activation of FAK can stimulate several signal transduction pathways such as Ras/MEK/ERK and PI3K/Akt pathways, leading to the activation or overexpression of downstream metastatic regulatory proteins^6^,^7^. Overexpression and hyperphosphorylation of FAK are associated with many types of solid tumors, which implying that the inhibition of FAK-stimulated tumor metastases by small molecule can provide a novel strategy for the development of therapeutic agents in tumor growth and metastasis^7^. Thus, it is badly needed to identify promising agents to suppress the activation of FAK and its downstream signaling pathways.

Ruthenium (Ru) complexes have attracted much attention as building blocks for novel metal-based anticancer and antimetastatic drugs since the discovery of cisplatin and the successful application of other platinum (Pt) complexes^8^,^9^,^10^. During the past three decades, a large number of potential Ru complexes have exhibited utility in chemotherapy, and some of them, including NAMI-A and KP1019, have entered into human clinical trials^8^,^11^. Various molecular mechanisms such as metastasis inhibition, interaction with DNA and induction of cell apoptosis, have been demonstrated as the anticancer action of Ru complexes. Accumulative evidences suggest that Ru-based compounds could act as potential antimetastatic agents for the treatment of human metastatic cancers^12^,^13^,^14^. For instance, NAMI-A, as a well-studied Ru(III) complex, has been shown to significantly suppress the formation of tumour metastasis in various animal models, which was associated with its general antimetastatic activity^11^,^15^. Anderson et al found that Hetero-multinuclear Ru(III)/Pt(II) complex, AH197, was much more efficient in the inhibition of cell motility than NAMI-A^16^. Nevertheless, the underlying mechanisms of the antimetastatic potential of these Ru-based agents are not still completely understood.

Ru polypyridyl complex is one type of metal-based complexes with potent anticancer activities^17^,^18^,^19^. In our previous work, a series of Ru polypyridyl complexes containing N,N-chelating ligands, such as 2,2-bipyridine (bpy), 4,4-dimethyl-2,2-bipyridine (dmb) and 1,10-phenanthroline (phen) have been prepared and evaluated for their anticancer activities^18^. The anticancer activities of phen complexes were higher than those of dmb and bpy complexes^18^. Among them, [Ru(phen)_2_-*p*-MOPIP](PF_6_)_2_·2H_2_O (RuPOP), as the most active phen complex, with-OCH_3_ on the p-site substitution, is able to induce caspase-mediated apoptosis in human melanoma A375 cells through the activation of mitochondrial signaling pathway^18^. However, many aspects of the inhibitory effects of RuPOP to tumor cells are not still completely understood. In the present work, RuPOP has been identified as a potent antimetastatic agent and metal-based chemosensitizer of TRAIL towards MDA-MB-231 human TNBC cells. The underlying mechanisms through which they caused the inhibition of metastatic potential of cancer cells and the promotion of TRAIL-induced apoptotic cell death were also clarified.

## Results

### Inhibition of cancer cell growth and metastasis by RuPOP *in vitro*

**Figure 1:**
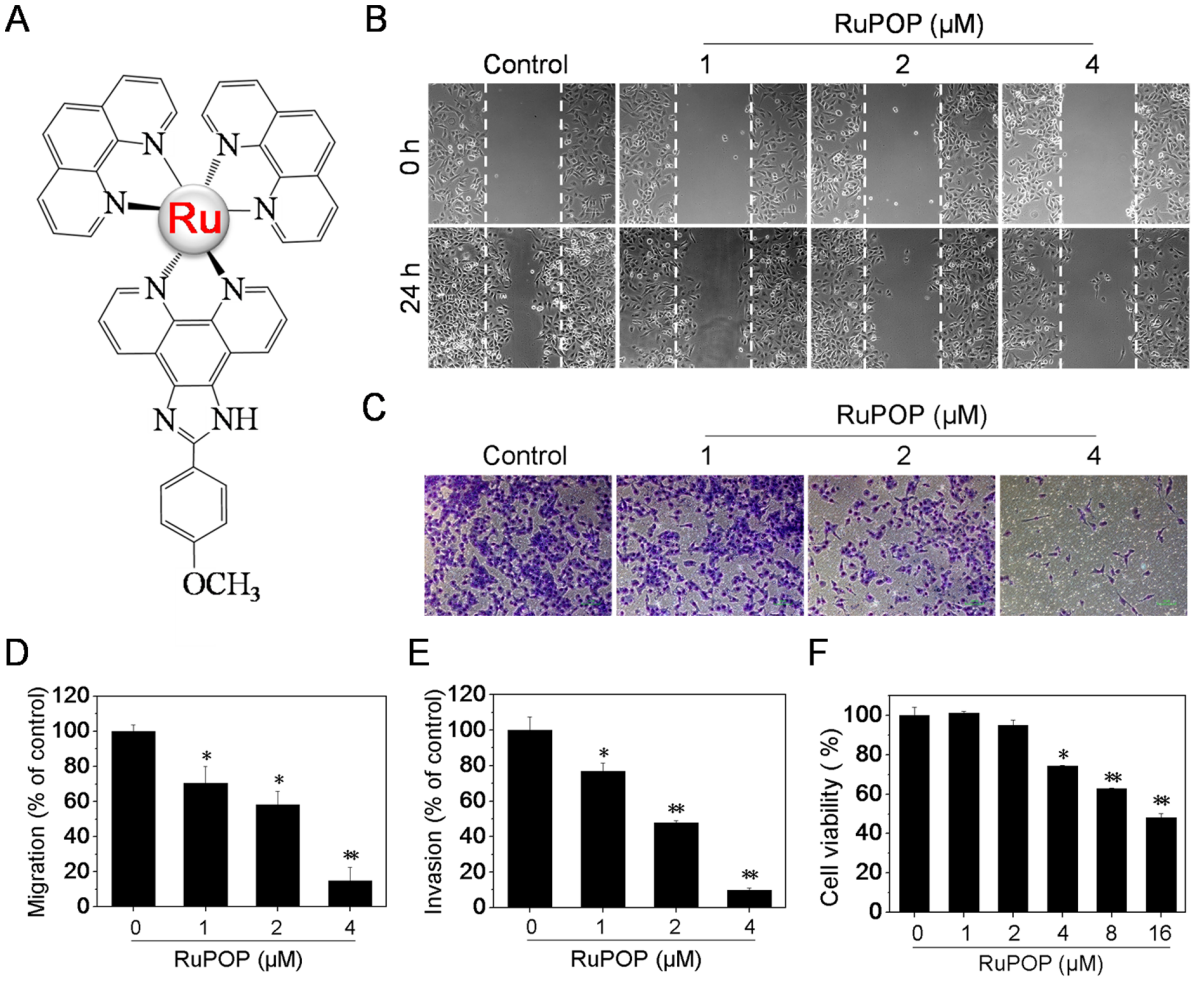
RuPOP inhibits growth and metastatic potential of human breast cancer cells *in vitro*. (A) The chemical structure of RuPOP. (B, C) Effects of RuPOP on the migration and invasion of MDA-MB-231 cells. Cells were exposed to different concentrations of RuPOP for 24 h and then photographed by a phase-contrast microscope (200×, Nikon TS100). (D, E) The migrated and invaded cells were quantified by manual counting and inhibition ratio was expressed as % of control. Each value represents the mean ± SD of three independent experiments, *, *P* < 0.05; **, *P* < 0.01 versus the control. (F) Cytotoxic effects of RuPOP on MDA-MB-231 cells. Cells were treated with indicated concentrations of RuPOP for 24 h.

The treatment of breast cancer is a clinically daunting task due to the diverse nature of the multiple breast cancer subtypes that each respond differently to the chemotherapeutic agents. Several Ru-based drugs have demonstrated promise in pre-clinical at inhibiting growth and metastatic potential of select subtypes of breast cancer^14^,^16^,^20^. In the present work, the growth inhibitory effects of RuPOP (Fig. 1A) *in vitro* was analyzed by MTT assay against four human breast cancer cell lines and a normal human kidney HK-2 cell line. As shown in Table 1, the diverse inhibition on the growth of the four tested human breast cancer cell lines induced by the synthetic RuPOP was observed, with IC_50_ values ranging from 14.6 μM to 78 μM after a 24 h treatment. In consistent with our previous study^18^, the cytotoxicity of RuPOP towards HK-2 cells was lower by comparing with cancer cell lines, with IC_50_ values at 143.9 μM, which indicates that RuPOP possesses selectivity between normal and cancer cells.

Interestingly, the four tested human breast cancer cell lines exhibited different sensitivity towards RuPOP. Among them, TNBC MDA-MB-231 cells, as a particularly aggressive cell line, exhibited the highest sensitivity to RuPOP. Due to the notable invasive properties of TNBC cells, we evaluated the effects of RuPOP on the metastatic potential of MDA-MB-231 cells *in vitro*. Scratch motility and transwell invasive assays were carried out to detect whether RuPOP could inhibit the metastatic potential of MDA-MB-231 cells. Results in Fig. 1B, C showed that RuPOP effectively inhibited the migration and invasion of MDA-MB-231 cells at the concentration of 1–4 μM after 24 h treatment. The migrated and invaded cells were quantified by manual counting and inhibition ratio was expressed as % of control (Fig. 1D, E). To confirm the observed suppression of migration and invasion were not due to the cytotoxic effect of RuPOP, the level of toxicity of RuPOP was measured at the indicated concentrations in Fig. 1F. RuPOP showed no obvious growth inhibitory effect at the concentration of 1–2 μM but slight cytotoxicity at the concentration of 4 μM after 24-h treatment, indicating that low-dose and short-term treatment of RuPOP blocks the migration and invasion of MDA-MB-231 cells in a cytotoxicity-independent manner.

### TfR-mediated selective cellular uptake of RuPOP

**Figure 2:**
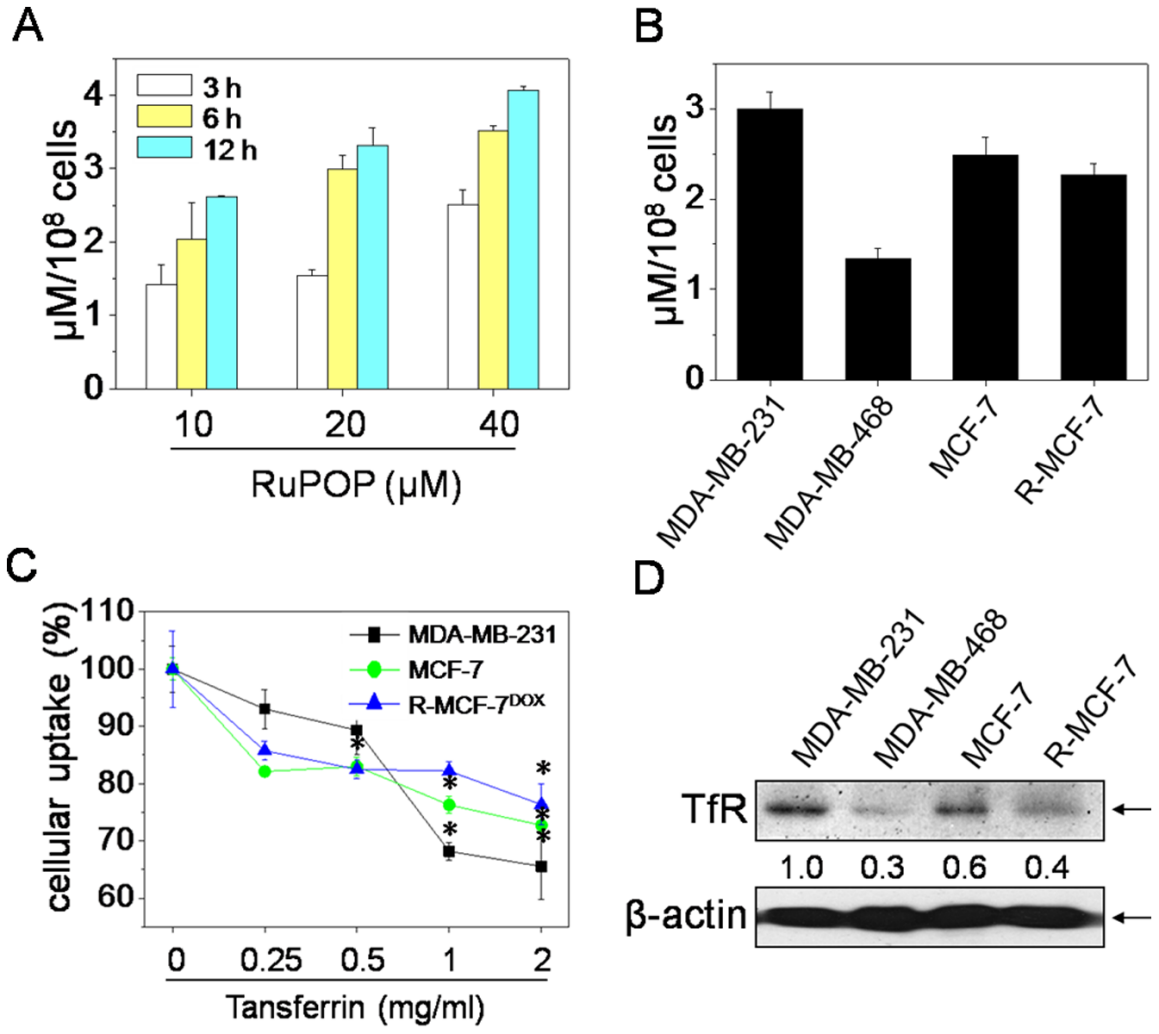
Selective cellular uptake of RuPOP in human breast cancer cells. (A) Quantitative analysis of cellular uptake efficacy of RuPOP in MDA-MB-231 cells. Cells were exposed to different concentrations of RuPOP for 3, 6 and 12 h respectively. (B) Quantitative analysis of cellular uptake efficacy of RuPOP in a panel of human breast cancer cell lines. Cells were treated with 20 μM of RuPOP for 6 h. (C) Inhibitory effects of Tf on cellular uptake of RuPOP in breast cancer cells. Cells were pretreated with indicated concentrations of Tf for 1 h and co-treated with 20 μM of RuPOP for 6 h. (D) Western blot analysis of the levels of TfR in the indicated human breast cancer cell lines. Changes in the levels of protein expression were shown as ratios of selected groups. The full-length blots/gels are presented in Supplementary Fig. S3. Each value represents the mean ± SD of three independent experiments, *, *P* < 0.05 versus the control.

The efficacy of cellular uptake is a critical factor that affects the metal-based agent activities and determines their successful clinical application. To clarify whether the different sensitivity of the tested breast cancer cell lines towards RuPOP is related to their cellular uptake efficacy, we compared the internalization of RuPOP in these cancer cell lines. Due to he strong autofluorescence of RuPOP, the quantitative analysis of cellular uptake was performed by measuring fluorescence intensity of RuPOP. As shown in Fig. 2A, the accumulation of RuPOP in MDA-MB-231 cells increased in a time- and dose-dependent manner. In addition, results in Fig. 2B showed that a clear divergence in RuPOP internalization in the four tested breast cancer cell lines was observed after incubation with 20 μM of RuPOP for 6 h. The efficacy of RuPOP internalization in the tested cell lines was as below: MDA-MB-231 > MCF-7 > R-MCF-7^DOX^ > MDA-MB-468, which was positive related to the growth inhibitor rate of RuPOP, suggesting that the internalization efficacy of RuPOP determine the anticancer activities of RuPOP towards the four tested breast cancer cell lines.

Cellular uptake is a complicated biological process that associates with the interaction between extracellular materials and the plasma membrane or the member receptor proteins. Transferrin receptor (TfR) is a well-known transmembrane glycoprotein that is overexpressed on the membrane of many cancer cells. TfR-directed targeting cellular uptake has been regarded as an efficient way for delivery of metal-based agents, such as Fe(III), Ru(III) and Ru(II) complexes to malignant tissues^20^. Guo et al found that Tf/TfR serves as a mediator enhanced the deliver of organometallic Ru(II) complexes into tumor cells^20^. Our previous work also demonstrated that the intracellular uptake of mixed-ligand Ru(II) polypyridyl complex ([Ru(bbp)(*o*-opip)]^2+^) was significantly blocked by pretreatment of TfR antibody^21^. Thus, we speculate that the selective intracellular uptake of RuPOP in the four tested breast caner cell lines may associate with their diversified transferrin receptor (TfR) expression profiles. To confirm this hypothesis, transferrin competing assay was used to detect the role of TfR in the cellular uptake of RuPOP. Results in Fig. 2C showed that pretreatment of transferrin effectively blocked the internalization of RuPOP in a does-dependent manner in three tested breast cancer cell lines. In addition, the expression level of TfR in the tested cell lines (Fig. 2D), as we expected, was positive related to the cellular uptake of RuPOP (Fig. 2B). These results demonstrate that the selective cellular uptake of RuPOP in the tested breast cancer cell lines may attribute to TfR-mediated endocytosis.

### Endocytosis and intracellular localization of RuPOP in MDA-MB-231 cells

**Figure 3:**
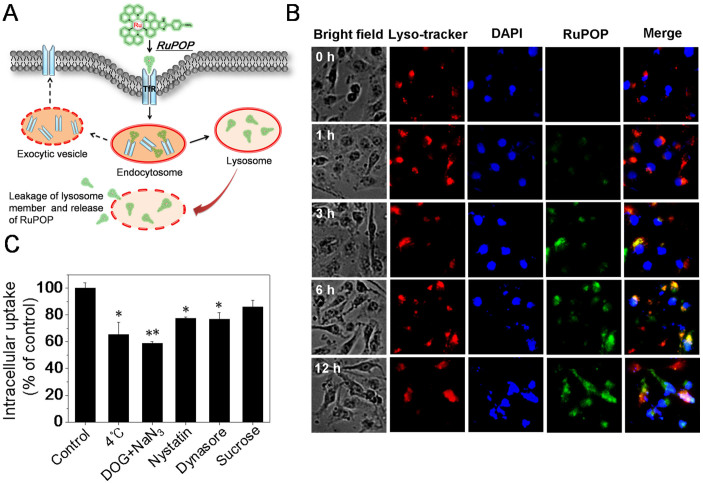
Endocytosis and intracellular localization of RuPOP in MDA-MB-231 cells. (A) Proposed endocytosis pathway of RuPOP in MCF-7 cells. (B) Intracellular tracking of RuPOP in MDA-MB-231 cells. Cells were exposed to 20 μM of RuPOP (green fluorescence) for the indicated time and co-stained with DAPI (blue fluorescence) and lyso-tracker (red fluorescence). (C) Detection of cellular uptake pathways for RuPOP (20 μM, 6 h) in MDA-MB-231 cells using specific endocytosis inhibitors (37°C) and lower temperature (4°C) treatment respectively. Each value represents the mean ± SD of three independent experiments, *, *P* < 0.05; **, *P* < 0.01 versus the control.

Generally, the cellular uptake of small molecules can occur via two ways, energy-dependent (such as endocytosis) and energy-independent (facilitated diffusion)^22^. As one of the most important entry mechanisms, endocytosis can regulate the biodistribution of extracellular molecular^22^,^23^. Based on the above evidences, we speculate that RuPOP may enter cells via TfR-mediated endocytosis (Fig. 3A). To further confirm this hypothesis, in the present study, two specific probes, DAPI and Lyso Tracker Red were used to investigate the intracellular location of RuPOP in MDA-MB-231 cells through fluorescence imaging of cell nucleus and lysosomes, respectively. As shown in Fig. 3B, the overlap of green and red fluorescence clearly demonstrated the colocalization of RuPOP and lysosomes, indicating that RuPOP moved across the cell member through endocytosis and lysosomes formation. According to the results of time-course analysis, we found that RuPOP entered in cells and accumulated in lysosomes in 3 h, followed by diffused into the whole cytoplasm from 6-h to 12-h treatment.

To clarify the cellular uptake mechanism of RuPOP, cells were pretreated with different endocytosis inhibitors before the incubation of RuPOP. Results in Fig. 3C showed that pretreatment of 2-deoxy-D-glucose (DOG) in combination with sodium azide (NaN_3_), or low temperature (4°C) pretreatment, significantly blocked the cellular uptake of RuPOP from 100% to 65.4% and 58.8%, indicating that internalization process is energy-dependent endocytosis. Clathrin-mediated, lipid raft/caveolae-mediated and phagocytosis/macropinocytosis represent three main modes of endocytosis^23^. Due to MDA-MB-231 cells are known to be not phagocytic, two inhibitors of lipid raft-mediated endocytosis (nystatin, dynasore) and a clathrin-mediated endocytosis inhibitor (sucrose) were used to investigate the major mode of endocytosis induced by RuPOP. As shown in Fig. 3C, the internalization of RuPOP was moderately decreased by nystatin, while slightly reduced by sucrose, indicating that lipid raft-mediated endocytosis is the main pathway of RuPOP internalization. As a GTP- dependent enzyme, dynamin is essential for receptor-mediated endocytosis^24^. dynasore, a specific inhibitor of dynamin-mediated lipid-raft endocytosis^25^, also moderately decreased internalization of RuPOP, suggesting that the dynamin-mediated pathway represents the major mechanism for lipid raft-dependent endocytosis in MDA-MB-231 cells.

### Critical roles of FAK-regulated ERK and Akt signaling pathways in the anticancer action of RuPOP

**Figure 4:**
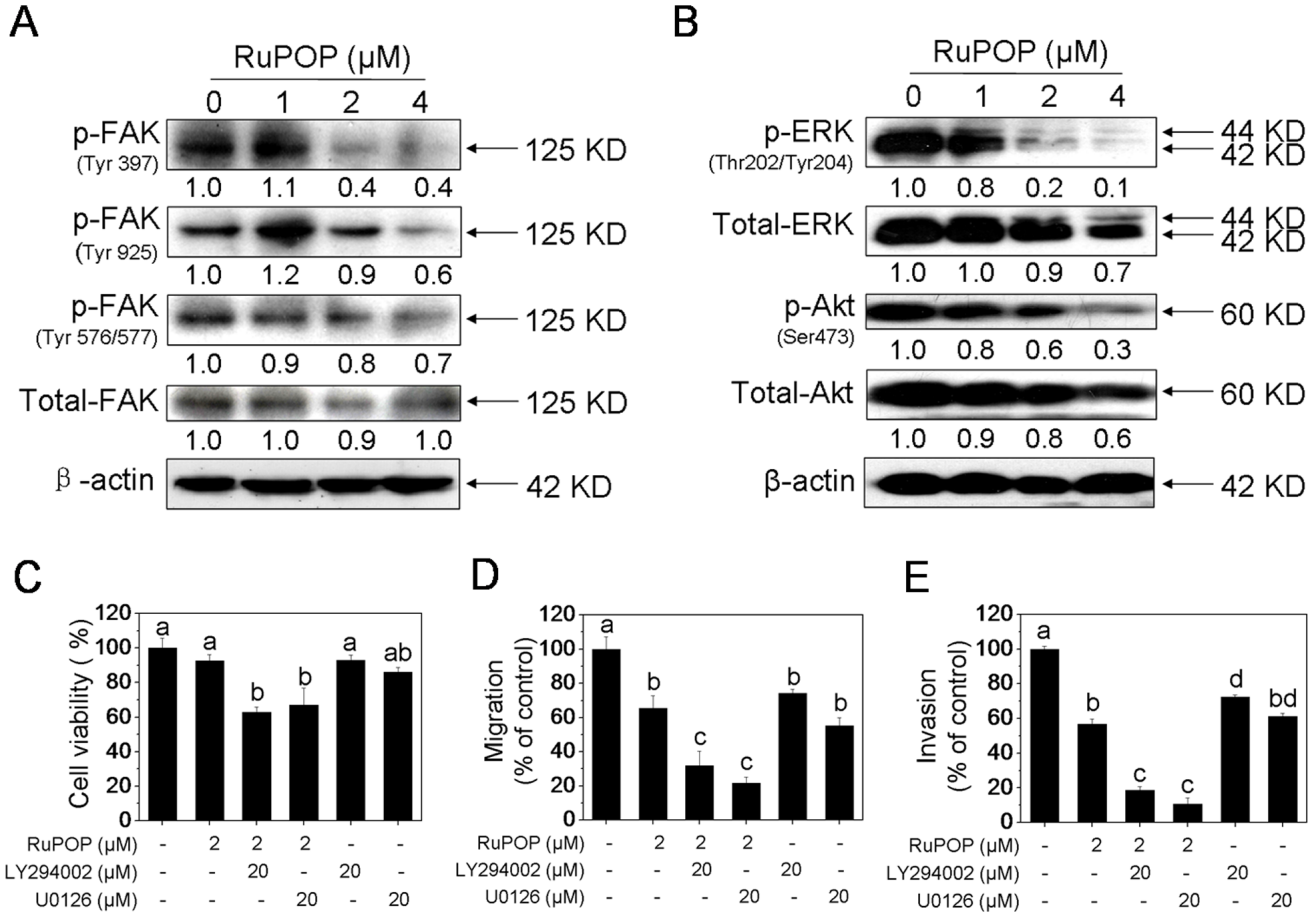
Suppression of FAK-mediated ERK and Akt posphorylation induced by RuPOP. (A, B) Western blot analysis the effects of RuPOP on the expression levels of phosphorylated and total FAK, ERK and Akt. Cells were exposed to different concentrations of RuPOP for 24 h. The changes in the levels of protein expression were exhibited as ratios of each group. The full-length blots/gels are presented in Supplementary Fig. S4, S5. (C–E) Effects of LY294002 and U0126 on RuPOP-induced inhibition on the growth, migration and invasion of MDA-MB-231 cells. Cells were pretreated with 20 μM LY294002 or U0126 for 1 h and co-treated with RuPOP for another 24 h. All data are expressed as means ± SD of triplicates. Bars with different characters (a, b, c and d) are statistically different at *P* < 0.05 level.

FAK, a cytoplasmic protein tyrosine kinase, plays a vital role in tumor cell proliferation, survival and migration, which associated with integrin-mediated signal transduction. Integrin clustering-mediated the activation of FAK results in the phosphorylation of Tyr397, which is a binding site for PI3K^26^. Recruitment of Src family kinases brings about the phosphorylation of Tyr871 and Tyr925 in the carboxy-terminal region and Tyr576/577 in the catalytic domain of FAK^27^,^28^. To illustrate weather the inactivation of FAK was involved in RuPOP-mediated inhibition of tumor cell growth and metastatic potential, we determined the levels of phosphorylated and total FAK in MDA-MB-231 cells after RuPOP treatment. Fig. 4A showed that RuPOP treatment significantly suppressed the expression levels of phosphorylated FAK at the site of Tyr397 and Tyr925, moderately inhibited the level of phosphorylated FAK at the site of Tyr576/577, while showed little effect on the level of total FAK. The results demonstrated that the inhibitory effect of RuPOP on MDA-MB-231 cells growth, migration and invasion may associate with the inactivation of FAK.

Studies have demonstrated that the activation of FAK by integrin engagement or growth factor stimulation promote the activation of downstream signaling transduction cascades including Ras/MEK/ERK and PI3K/Akt, which can positive regulate pro-metastatic proteins^6^,^7^,^28^. Thus, in the present study, we examined whether ERK and Akt were inactivated in RuPOP-treated MDA-MB-231 cells by Western blot analysis. It was found that RuPOP treatment markedly decreased the expression levels of phosphorylated ERK and Akt and moderately decreased total ERK and Akt in MDA-MB-231 cells (Fig. 4B). In addition, LY294002 (PI3K inhibitor) and U0126 (ERK inhibitor) were used to evaluate whether suppression of Akt and ERK related signaling pathways were needed in RuPOP-induced cell growth and metastatic potential inhibition. As shown in Fig. 4C-E and Supplementary Fig. S2, treatment with 20 μM of LY294002 and U0126 or 2 μM of RuPOP alone for 24 h showed no obvious inhibitory effects on cell growth but notable inhibitory effects on the migration and invasion of MDA-MB-231 cells. Pretreatment with 20 μM of LY294002 or U0126 for 1 h significantly decreased the proportion of growth, migration and invasion of MDA-MB-231 cells by comparing with the single treatment. These results demonstrated that inactivation of Akt and ERK signaling was involved in RuPOP-induced cell growth and metastatic potential inhibition in MDA-MB-231 cells. On the basis of above evidences, we suggest that RuPOP inhibits growth and metastatic potential of MDA-MB-231 cells via suppression of FAK-mediated ERK and Akt activation.

### RuPOP alternates the expression levels of metastatic regulatory proteins and inhibits the secretion of VEGF

**Figure 5:**
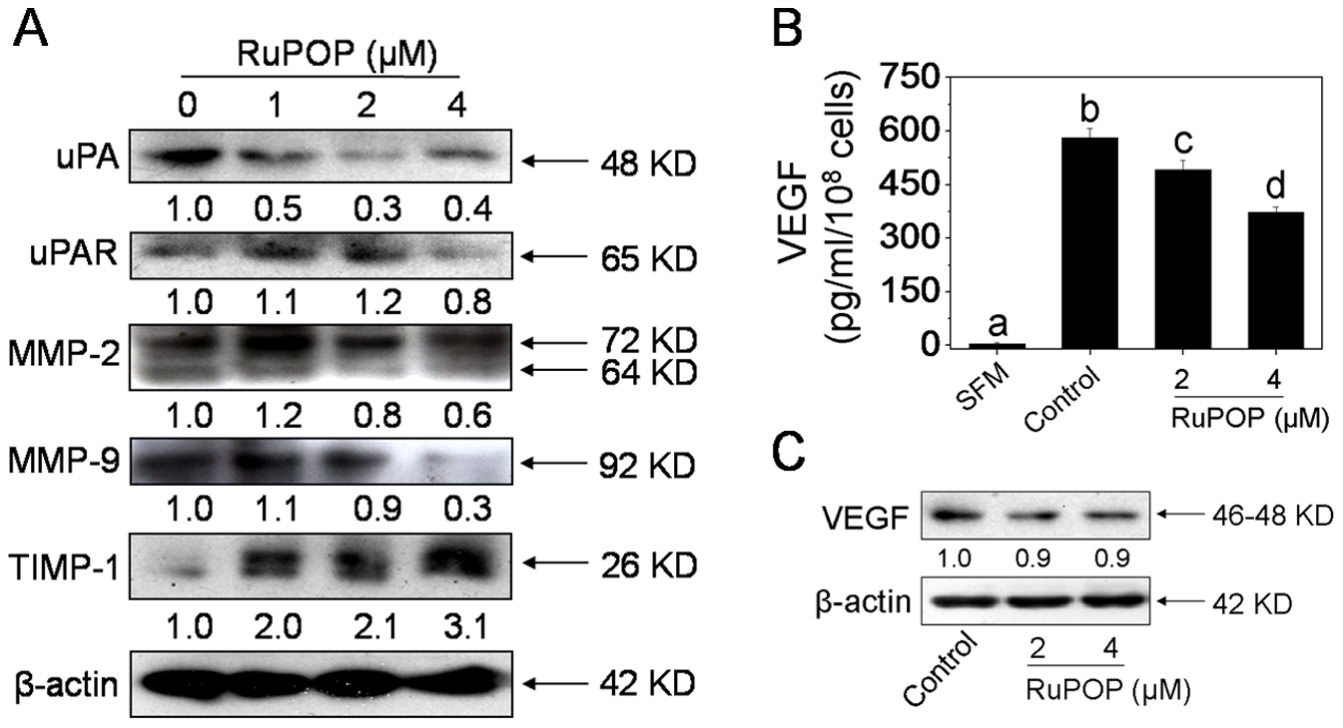
RuPOP regulates the expression levels of MMPs and its regulatory proteins and blocks the secretion of VEGF in MDA-MB-231 cells. (A) Western blot analysis the effects of RuPOP on the expression levels of uPA, uPAR, MMP-2, MMP-9 and TIMP-1. Cells were exposed to indicated concentrations of RuPOP for 24 h. Changes in the levels of protein expression were shown as ratios of selected groups. (B, C) Effects of RuPOP on the expression and secretion of intracellular VEGF. Cells were exposed to different concentrations of RuPOP for 24 h. The full-length blots/gels are presented in Supplementary Figs. 6, 7. All data are expressed as means ± SD of triplicates. Bars with different characters (a, b, c and d) are statistically different at *P* < 0.05 level.

The levels of MMPs and uPA are known to associate with tumor invasion and metastasis. Activated FAK can stimulate Ras/MEK/ERK and PI3K/Akt pathways, leading to the activation or overexpression of MMPs and uPA^6^,^7^. MMPs play critical roles in normal tissue remodeling in many biological process, such as embryonic development, tumor invasion, carcinogenesis and apoptosis^5^. Among the family members, MMP-2 and MMP-9 were overexpressed in the uterus and breast of humans and may contribute to the invasion of cancer^29^. Studies have demonstrated that Ru complexes could effectively inhibit the activation or expression of MMP-2/-9^13^,^30^. On the basis of above evidences, we thus speculate that the MMPs and its positive regulator uPA, as the downstream metastatic regulatory proteins of FAK-mediated signaling pathways, were also regulated after the RuPOP treatment. To verify the hypothesis, western blot analysis was used to determine the expression levels of downstream metastatic regulatory proteins, including uPA, uPAR and MMP-2/-9. Results in Fig. 5A showed that RuPOP treatment significantly down-regulated the levels of uPA and MMP-9, up-regulated the levels of TIMP-1 (a tissue inhibitor of metalloproteinases) and moderately decreased the levels of MMP-2.

In addition, as a critical mediator of tumor angiogenesis, vascular endothelial growth factor (VEGF) highly secreted in invasive cancer cells and could regulate proliferation, migration, and tube formation of endothelial cells^31^. Studies have demonstrated that MMP-9 and uPA, as the matrix-degrading protease and key serine protease respectively, were able to promote the degradation of ECM, leading to the release of bound-VEGF into tissue culture media^32^. In order to further confirm whether the secretion of VEGF was blocked, we analyzed the levels of VEGF in conditioned media from MDA-MB-231 cells incubated with different concentrations of RuPOP. As shown in Fig. 5B, invasive breast cancer MDA-MB-231 cells secreted high levels of VEGF by comparing with the group of free serum-free medium (SFM). RuPOP treatment obviously decreased the VEGF secretion from MDA-MB-231 cells. Furthermore, no significant alternations were found in the expression level of VEGF after RuPOP treatment (Fig. 5C). Therefore, we speculated that down-regulated secretion of VEGF in culture media from MDA-MB-231 cells treated with RuPOP may correlate with the decreased the expression levels of uPA, MMP-2 and MMP-9 and increased the expression level of TIMP-1, which means RuPOP treatment reduced the bioavailability of bound-VEGF. Taken together, these data demonstrate that RuPOP inhibits the metastatic potential and VEGF secretion of MDA-MB-231 cells though regulating the expression levels of metastatic proteins.

### RuPOP synergistically enhances the anticancer efficacy of TRAIL

Tumor necrosis factor–related apoptosis-inducing ligand (TRAIL), as a potent targeted drug, has received a great deal of attention due to it induces apoptosis in various tumor cells but not in normal cells^33^,^34^. In addition to triggering a death receptor-mediated pro-apoptotic signaling pathway, TRAIL treatment can also activate intracellular pro-survival NF-κβ^35^, PI3K/Akt^36^,^37^ and ERK^38^ signaling pathways, which indicates that TRAIL treatment alone may be insufficient for cancer therapy. Therefore, agents are urgently needed that can sensitize the cancer cells to TRAIL. Study has suggested that the combination treatment of metal-based agent (cisplatin) and TRAIL have the potential of providing a novel strategy for improving the chemotherapeutic efficacy in TNBC patients^39^. Recently, we also showed that, pretreatment of cells with organic selenium compound could enhance the apoptosis-inducing efficacy of TRAIL in A375 cells through induction of ROS-dependent Akt dephosphorylation^40^. Results in the present work showed that RuPOP effectively suppressed FAK-mediated ERK and Akt phosphorylation after 24 h treatment in MDA-MB-231 cells (Fig. 4). On the basis of these evidences, we predict that RuPOP could act as a chemo-sensitizer to enhance TRAIL-induced apoptosis in MDA-MB-231 cells.

**Figure 6:**
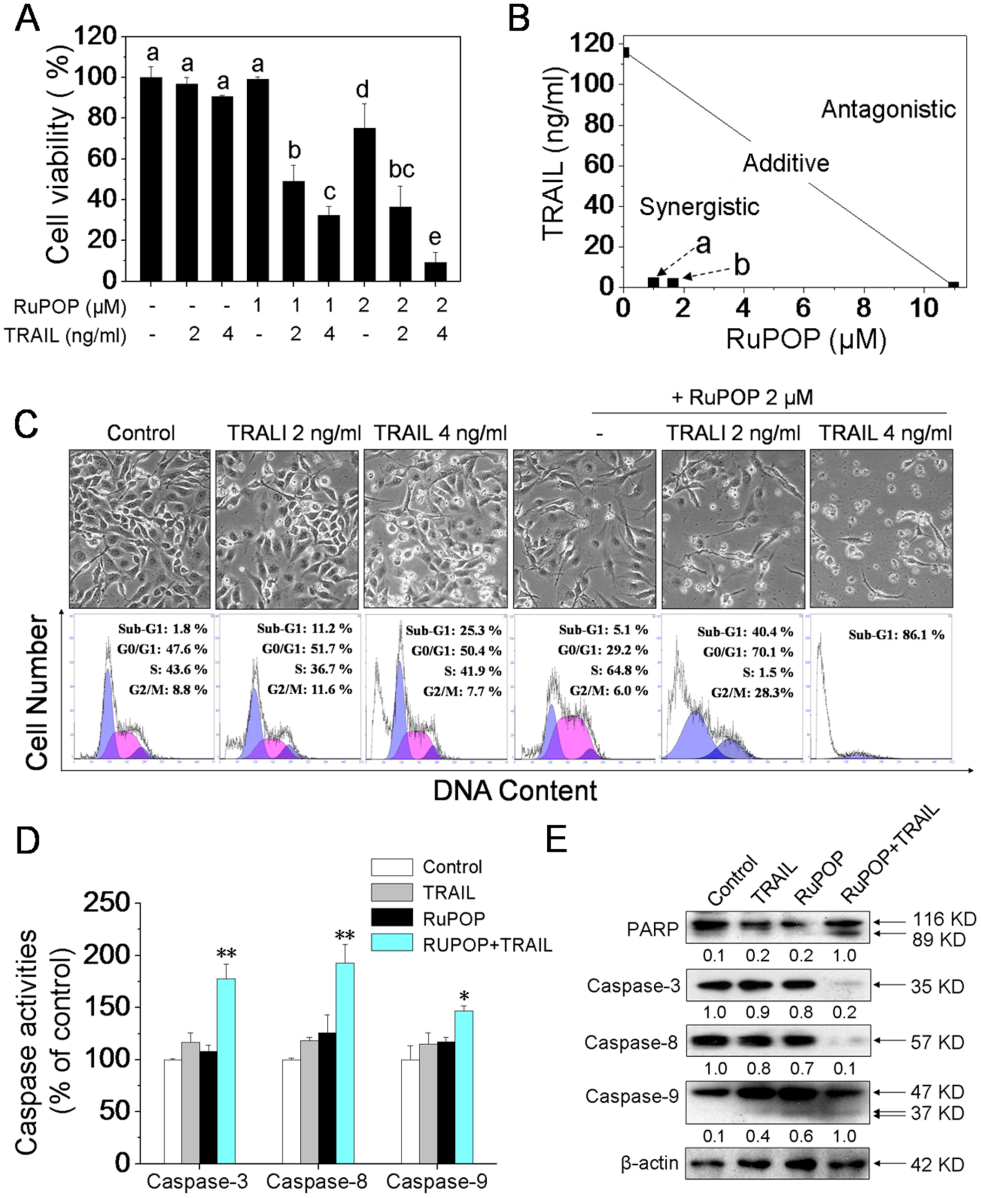
RuPOP synergistically enhances the anticancer efficacy of TRAIL through activation of intrinsic and extrinsic apoptotic pathways. (A) RuPOP enhances the efficacy of TRAIL-induced MDA-MB-231 cells growth inhibition. Cells were pretreated with or without indicated concentrations of RuPOP for 24 h and then co-treated with 2, 4 ng/ml TRAIL for another 24 h. Each value represents the mean ± SD of three independent experiments. Bars with different characters (a, b, c, d and e) are statistically different at *P* < 0.05 level. (B) Isobologram analysis of the synergistic anticancer effect of co-treatment on MDA-MB-231 cells. The data points (a, b) in the isobologram correspond to the actual IC_50_ value of RuPOP and TRAIL with different ratio of concentrations in the co-treatment. RuPOP (μM) : TRAIL (ng/ml) = 1:2, 1:1. (C) Quantitative analysis of cell cycle distribution and apoptotic cell death were measured by flow cytometric analysis. Cells were pretreated with or without 2 μM RuPOP for 24 h and then exposed to 2, 4 ng/ml TRAIL for another 24 h. (D) Detection of caspase activation induced by RuPOP (2 μM) and/or TRAIL (2 ng/ml). Each value represents the mean ± SD of three independent experiments, *, *P* < 0.05; **, *P* < 0.01 versus the control. (E) Western blot analysis of expression levels of PARP and caspase family members in MDA-MB-231 cells exposed to 2 μM RuPOP and/or 2 ng/ml TRAIL. The changes in the levels of protein expression were exhibited as ratios of each group. The full-length blots/gels are presented in Supplementary Fig. S8.

To further confirm this hypothesis, MTT assay was carried out to evaluate the synergistic anticancer effects of RuPOP and/or TRAIL treatment. Firstly, the treatment of MDA-MB-231 cells with 0–16 μM of RuPOP for 48 h or 0–400 ng/ml TRAIL for 24 h suppressed cell growth in a dose-dependent manner (Supplementary Fig. S1A, B). In order to establish an optimal strategy in the combined treatment, cells were pretreated with different concentrations of RuPOP for 0, 12 and 24 h, and followed by co-treatment of indicated concentrations of TRAIL for additional 24 h. As shown in Fig. S1C and Fig. 6A, pretreatment of cells with 1, 2 μM RuPOP for 24 h and then co-treatment with 2, 4 ng/ml TRAIL for 24 h significantly inhibits cell growth, indicating that the RuPOP pretreatment notably enhances the growth inhibitory efficacy of TRAIL in a dose- and time-dependent manner. Despite the notable growth inhibitory effects, the combined treatment with RuPOP (20 μM) and TRAIL (40 ng/ml) exhibited lower cytotoxicity towards human normal cell lines HK-2 (Fig. S1D).

To determine the interaction between RuPOP and TRAIL, the growth inhibition of individual and combined treatments was evaluated by isobologram analysis^41^. The growth inhibitory potential of co-treatment of RuPOP and TRAIL under different ratios (1:2 and 1:1) were found to be statistically synergistic, as evidenced by the site of the points in isobologram being far below from the line defining additive effect (Fig. 6B). In addition, the combination index (CI) of the co-treatments were calculated at 0.11 (1:2) and 0.16 (1:1), which further confirmed the significant synergistic effects between RuPOP and TRAIL. Therefore, our data demonstrate that RuPOP could act as an efficient agent to enhance TRAIL-induced apoptosis in MDA-MB-231 cells.

### RuPOP potentiates TRAIL-induced apoptosis though intrinsic and extrinsic apoptotic pathways

Apoptosis is a critical mechanism for chemoprevention and chemotherapy of various cancer cells^42^. TRAIL, as one of the potent targeted anticancer agent, could selectively kill a variety of cancer cells through activation of death receptor-mediated apoptosis pathway^33^. Our previous studies also found that Ru polypyridyl complexes could inhibit cancer cells growth by triggering distinct apoptosis signaling pathways^18^,^19^,^21^. Therefore, to elaborate whether the apoptosis-inducing efficacy of TRAIL was enhanced by RuPOP, cells after treatments were determined by flow cytometric analysis to examine the effects on apoptotic cell death and cell cycle progression. Results in Fig. 6C showed that exposure of MDA-MB-231 cells to RuPOP and/or TRAIL resulted in marked increases in the number of apoptotic cells as evidenced by the peaks of sub-diploid. For instance, the sub-G1 population was slightly alternated after TRAIL treatment alone, whereas significantly enhanced from 11.2% and 25.3% to 40.4% and 86.1% after co-treatment with 2 μM RuPOP and 2, 4 ng/ml TRAIL. However, no significant changes in the accumulation of Sub-G1 population but obvious S-phase arrest were observed after RuPOP treatment alone. These data suggest that apoptosis may be the major mechanism of cell death triggered by the co-treatment.

Caspases is important class of cysteine acid proteases that play an important roles in control of cell apoptosis by activation of various cellular substrates^43^. In order to further prove the above findings, we investigated the requirement of caspases for the RuPOP and/or TRAIL treatment-mediated apoptotic program. The activity of caspase-3 and two initiator caspases, caspase-8 (death receptor pathway-related)/-9 (mitochondrial pathway-related) were detected by fluorometric assay. Results shown in Fig. 6D revealed a remarkably increase in caspase-3 activities induced by the co-treatment, which demonstrate that the combined treatment-induced growth inhibition is mainly attributed to the induction of apoptosis. Moreover, the co-treatment noticeable activated caspase-8/-9 in MDA-MB-231 cells (Fig. 6D), which indicate that both death receptor and mitochondrial-related apoptotic signaling pathways were involved in co-treatment-induced apoptosis. To further confirm these data, Western blot analysis was used to determine the activation of caspases and cleavage of PARP, a bio-marker of cells undergoing apoptosis. As shown in Fig. 6E, the co-treatment effectively triggered the activation of caspase-3/-8/-9 and PARP cleavage, which consistent with the results in Fig. 6D. Taken together, the above evidences suggest that RuPOP potentiated TRAIL-induced apoptosis though intrinsic and extrinsic apoptotic pathways.

## Discussion

As the end product of invasion-metastasis cascade, tumor metastases plays a critical role for causing death in cancer patients^4^. The metastatic potential of tumor cells is regulated by a number of the activated proteases, including uPA/uPAR system-mediated MMPs activation. Accumulative studies have demonstrated that Ru complexes, especially the well-studied NAMI-A, could effectively suppress the growth and metastatic potential of human metastatic cancer cells through modulating the activities and expression levels of MMP-2 and MMP-9 in a variety of tumour models^12^,^13^,^30^. Nevertheless, little information about the underlying mechanisms of inactivation or down-regulation of MMPs by these Ru complexes could be obtained. Thus, rationally, with the purpose to synthesize new metal-based antimetastatic drug leads, a better understanding of the physiological properties of metastasis is required.

Ru polypyridyl complexes, especially RuPOP, exhibited great cytotoxic effects against a panel of human cancer cell lines after a long-term (48 h) and high-dose treatment^18^. In consistent with the previous data, the results in the present study also demonstrated the cytotoxic effects of high concentration of RuPOP toward the tested human breast cancer cell lines (Fig. 1, Fig. S1 and Table 1). These data indicate that the cytotoxic effect is one of the major mechanisms of high-dose and long-term treatment of RuPOP in human cancer cells. However, in order to further explore the other anticancer action of RuPOP, we examined the antimetastatic potential of this complex in the present study. Interestingly, as shown in Fig. 1 and Fig. S2, RuPOP was identified as a potent antimetastatic agent and metal-based chemosensitizer of TRAIL towards MDA-MB-231 cells. Even under low concentration of 1–2 μM, RuPOP was able to effectively inhibit the metastatic potential of MDA-MB-231 cells with no obvious cytotoxic effects and synergistically enhance TRAIL-induced apoptosis. Furthermore, the underlying molecular mechanisms were also elucidated.

Cellular uptake is a complex process and refers to the interaction between RuPOP and the plasma membrane. A better understanding of cellular uptake mechanisms will promote the application of RuPOP. Puckett and Barton have demonstrated that Ru polypyridyl complex (Ru(DIP)_2_dppz^2+^) entered cells through passive diffusion^22^. Our previous study also showed that Ru(II) polypyridyl complexes entered cancer cells by a combination of endocytosis and passive diffusion pathways^21^. In the present study, the blocking effects of Tf on RuPOP internalization (Fig. 2) and colocalization of RuPOP and lysosomes were clearly observed (Fig. 3), indicating that RuPOP entered the cell member through receptor-mediated endocytosis and lysosomes formation. Furthermore, metabolic inhibition (Fig. 3C) significantly blocked the cellular uptake of RuPOP from 100% to 65.4% and 58.8%, indicating that internalization of RuPOP is energy-dependent process. Based on the above evidences, we suggest that TfR-mediated endocytosis is a major mechanism for RuPOP internalization.

FAK, as an upstream mediator in the cell signaling transduction pathways, plays a vital role in tumor cell proliferation, survival and migration. Clinical researches indicate that FAK-related changes of tumor metastasis are associated with increased risk of developing solid tumors^7^. Therefore, blocking FAK activation and its interactions with proteins can be an attractive approach to combat tumor growth and metastasis. Several drugs, such as sphingolipid analog FTY-720 and statins that either directly or indirectly affect the activities of FAK are developed in recent years^44^. Many of these agents have been studied in preclinical models yet seem to hold therapeutic promise. In the present study, we found that, the inactivation of FAK was involved in RuPOP-mediated inhibition of tumor cell growth and metastatic potential (Fig. 4). The downstream pro-survival signaling transduction pathways, Ras/MEK/ERK and PI3K/Akt were also inhibited by RuPOP, as evidenced by the obvious down-regulation of phosphorylated ERK and Akt, which alternate the expression levels of the metastatic regulatory proteins (Fig. 5). Based on the above data, we suggest that RuPOP inhibits growth and metastatic potential of MDA-MB-231 cells via suppression of FAK-mediated ERK and Akt activation.

**Figure 7:**
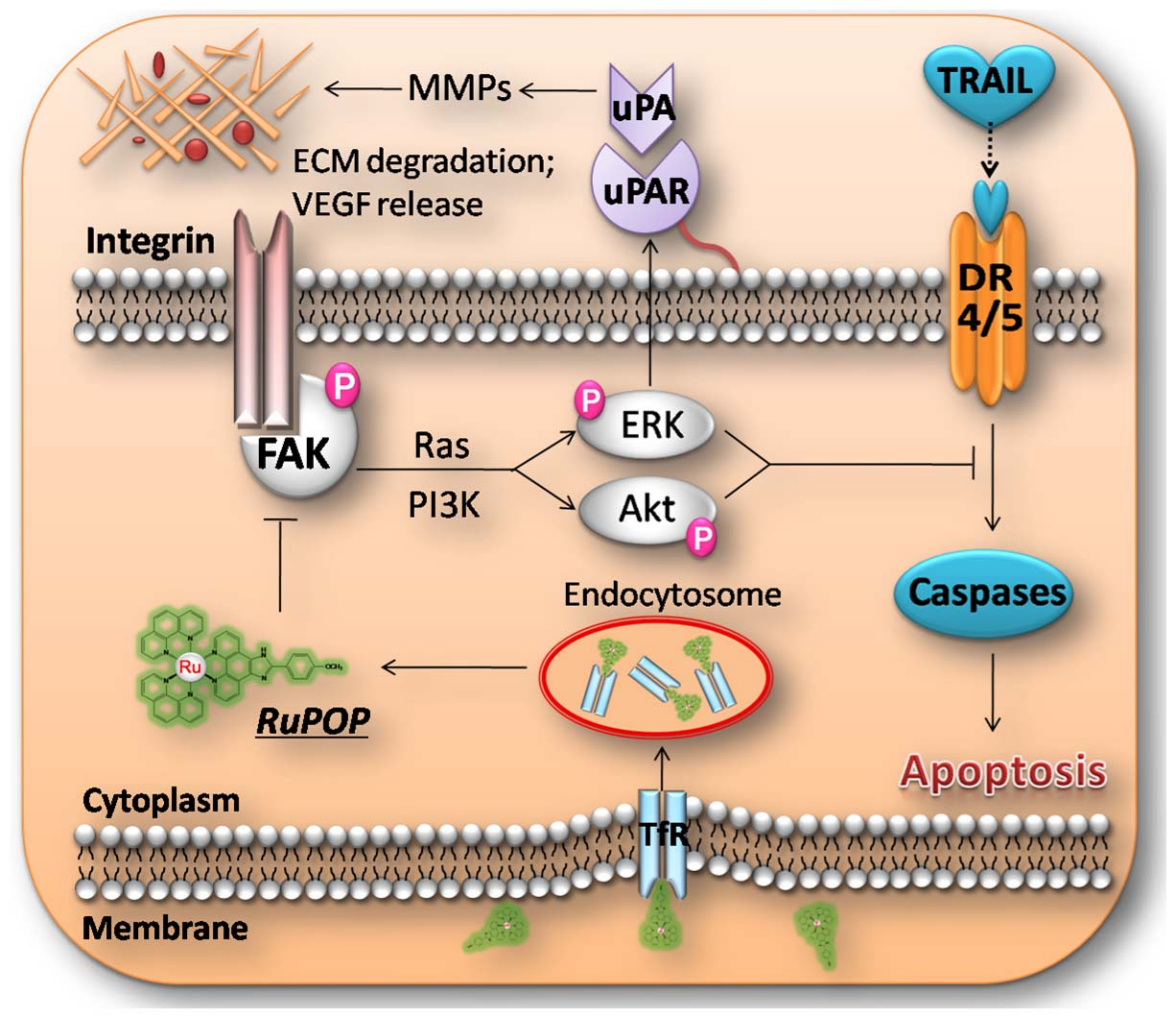
Proposed signaling pathway triggered by RuPOP and TRAIL in MDA-MB-231 cells. RuPOP entered into the cells through TfR-mediated endocytosis, accumulated in lysosomes in 3 h, and diffused in the cytoplasm from 6 h to 12 h. The internalized RuPOP suppressed FAK-mediated ERK and Akt activation, leading to the alternation of the expression levels of the downstream metastatic regulatory proteins, including uPA, uPAR, MMP-2, MMP-9 and TIMP-1. Down-regulation of the levels of the uPA and uPAR influenced the activation of plasminogen to plasmin and the cleavage of MMPs, resulting in the decrease of VEGF release. In addition, inactivation of ERK and Akt promoted TRAIL-induced apoptosis via activation of caspase cascade and cleavage of PARP.

In summary, Ru polypyridyl complex (RuPOP) has been identified as a potent antimetastatic agent and metal-based chemosensitizer towards MDA-MB-231 cells. The selective intracellular uptake and cytotoxic effect of RuPOP against four human breast cancer cell lines and a human normal kidney cell line HK-2 was associated with TfR-mediated endocytosis. Further determination on molecular mechanisms demonstrated that RuPOP was able to regulate the expression levels of metastatic regulatory proteins, inhibit the secretion of VEGF and potentiate TRAIL-induced apoptosis in MDA-MB-231 cells through inhibition of FAK-mediated ERK and Akt activation (Fig. 7). On the basis of these data, we suggest that the combined treatment of RuPOP with TRAIL could be a novel strategy to inhibit growth and metastatic potential of tumor cells and synergistically enhance TRAIL-induced apoptotic cell death.

## Methods

### Preparation of RuPOP

RuPOP was synthesized according to our previous methods^18^. Briefly, A mixture of cis-[Ru(phen)_2_Cl_2_]·2H_2_O and *p*-MOPIP in ethyleneglycol was refluxed under argon for 4 h to give a clear red solution. The crude product was purified by column chromatography on a neutral alumina. The mainly red band was collected. The solvent was removed under reduced pressure and an orange-red powder was obtained.

### Cell culture and MTT assay

Human breast adenocarcinoma MCF-7cells and normal human kidney HK-2 cells were purchased from ATCC (Manassas, VA). Human TNBC cells MDA-MB-231/-468 were obtained from kyGEN (Nanjing, China). The cells cultured in DMEM medium with fetal bovine serum (10%), streptomycin (50 units/ml) and penicillin (100 units/ml) in an incubator (5% CO_2_) at 37°C. The doxorubicin-resistant MCF-7 (R-MCF-7^DOX^) cell line was established by culturing MCF-7 cells in the above media with gradually increasing concentrations of doxorubicin (0.2–1 μg/ml) and then maintaining them in 1 μg/ml doxorubicin for ten months. The effects of RuPOP and/or TRAIL on the cell growth were determined by MTT (Sigma-Aldrich) assay. Briefly, cells were seeded in 96-well culture plates for 24 h and incubated with different concentrations of RuPOP or TRAIL for 24 or 48 h, Then, 20 μl per well of MTT solution (5 mg/ml) was added and incubated for 5 h. The medium was aspirated and replaced with 200 μl per well of DMSO to dissolve the formazan salt formed. The color intensity of the formazan solution was measured at 570 nm using a microplate spectrophotometer (VERSA max).

### Scratch motility (wound-healing) assay

MDA-MB-231 cells (8 × 10^4^ cells/ml) were cultured in 6-well plates and allowed to form a confluent monolayer for 24 h. After serum starved for 4 h, cells were scratched by pipette tips, washed with PBS and photographed by using a phase-contrast microscope (200×, Nikon TS100). The fresh medium supplemented with 1% FBS was added into well with different concentrations of RuPOP. After incubated for 24 and 48 h, cells were photographed again at three randomly area. Then the migrated cells were quantified by manual counting and inhibition ratio was expressed as % of control.

### Transwell invasion assay

Effects of RuPOP on the invasion of MDA-MB-231 cells were performed on Transwell Boyden chamber (8 μm pore, Corning, Lowel, MA) pre-coated with matrigel for 4 h at 37°C. 100 μl of cell suspension (2.5 × 10^5^ cells/ml) in serum free medium (SFM) was placed to the upper compartment of chamber. The bottom chambers were supplemented with 500 μl complete medium (10% FBS) containing indicated concentrations of RuPOP. After incubated for 24 h, the non-migrant cells from the upper face were scraped using a cotton swab. The invaded cells on the lower face were fixed with methanol, stained with Giemsa, photographed by a phase-contrast microscope (200×, Nikon TS100). The invaded cells were quantified by manual counting and inhibition ratio was expressed as % of control.

### Intracellular uptake of RuPOP

The intracellular uptake of RuPOP was determined by using a microplate spectrophotometer (Spectra Max M5, Bio-Tek) according to our previous methods^45^. Briefly, the treated cells were washed with PBS three times and lysed by 100 μl/well of Triton X-100(1%) in 0.1 M NaOH solution. Then the fluorescence intensity of the internalized RuPOP was detected with excitation and emission wavelengths set at 418 and 590 nm, respectively. The cell number was determined by Trypan blue staining assay, and thus the intracellular RuPOP content was calculated as μM RuPOP per 10^8^ cells.

### Transferrin blocking assay

Cells were cultured in 96-well plates for 24 h. Then 0.25–2 mg of transferrin (Sigma-Aldrich) were added to the wells for 1 h, followed by the co-treatment of RuPOP (20 μM) for 6 h. After that, the cells were washed with PBS and lysed by 100 ml/well of Triton X-100(1%) in 0.1 M NaOH solution, followed by the fluorescence intensity measurement of the internalized RuPOP (Spectra Max M5, Bio-Tek).

### Intracellular tracking of RuPOP

The intracellular localization of RuPOP in MDA-MB-231 cells was traced by Lyso-tracker red (Invitrogen) and DAPI (Sigma-Aldrich) co-staining. Briefly, the treated cells were washed and replaced with fresh serum-free culture medium. Then cells were stained with lyso-tracker red (1 μM) for 2 h and DAPI (1 μg/mL) for 30 min. After washing with PBS twice, the cells were visualized under a fluorescence microscope (200×, Life technologies EVOS® FL Auto).

### Mechanisms of cellular uptake of RuPOP

Cells were cultured in 96-well plates for 24 h. After the pretreatment with endocytosis inhibitors for 1 h, and then co-treatment with 20 μM of RuPOP for another 6 h, the fluorescence intensity of internalized RuPOP was measured according to the above method. The function and final concentrations of different endocytosis inhibitors were listed as below: 10 mM sodium azide (NaN_3_) (ATP synthesis inhibitor), 50 mM 2-deoxy-D-glucose (DOG) (ATP synthesis inhibitor), 0.45 M Sucrose (nonspecific clathrin inhibitor), 80 μM dynasore (a specific inhibitor of dynamin) and 10 μg/ml nystatin (lift-raft inhibitor)^25^,^46^. For investigation of energy-dependent pathways, the cells were incubated at Low temperature treatment (4°C) for 4 h, followed by the incubation of 20 μM of RuPOP for another 6 h.

### Determination of extracellular VEGF

The concentrations of extracellular VEGF was detected by using Quantikine® ELISA kit (R&D System). Briefly, cells (8 × 10^4^ cells/ml) were cultured in 6-well plate for 24 h, washed with PBS twice and replaced with fresh serum-free medium containing different concentrations of RuPOP. After 24 h treatment, the culture media were collected and centrifuged to eliminate cellular debris. Then, the collected medium was added into the detected microplate and incubated for 2 h at room temperature. After three washes, the VEGF conjugate was added and incubated for another 2 h. After another three washes and the addition of substrate solution and stop solution, the detection of VEGF concentration in the culture medium was performed by using a microplate spectrophotometer (VERSA max, Molecular Devices) set at 450 nm.

### Isobologram analysis

The synergistic effect between RuPOP and TRAIL was evaluated by the isobologram method^41^. Briefly, a straight line was formed by plotting the IC_50_ values of DSeA and TRAIL on the x- and y-axes, respectively. The data point in the isobologram corresponds to the actual IC_50_ value of RuPOP and TRAIL with different ratio of concentrations in the combined treatment. If a data point is on or near the line, which represents additive effect, whereas a data point that lies below or above the line indicates synergism or antagonism, respectively. The combination index (CI) was calculated to determine the interaction between RuPOP and TRAIL. CI value: <1.0, synergism; = 1.0, additive effect; >1.0, antagonism.

### Determination of cell apoptosis

The effects of RuPOP and/or TRAIL on the cell cycle progression and the induction of apoptotic cell death were quantified by flow cytometric analysis. Briefly, treated or untreated cells were trypsinized, washed with PBS and fixed with 70% ethanol overnight at −20°C. The fixed cells were washed with PBS and incubated with a PI working solution for 4 h in darkness. The stained cells were analyzed with flow cytometer (Beckman Coulter, Fullerton, CA). Cell cycle distribution was analyzed using MultiCycle software (Phoenix Flow Systems, San Diego, CA). The proportion of cells in G0/G1, S, and G2/M phases was represented as DNA histograms. Apoptotic cells with hypodiploid DNA content were measured by quantifying the sub-G1 peak in the cell cycle pattern. For each experiment, over 10000 events per sample were recorded.

Caspase activity was detected by fluorescence assay using specific substrates. Briefly, cell lysates were placed in 96-well plates and then the specific caspase substrates were added. Plates were incubated at 37°C for 2 h and caspase activity was determined by measuring the fluorescence intensity on a microplate spectrophotometer (Spectra Max M5, Bio-Tek) with the excitation and emission wavelengths set at 380 and 440 nm, respectively.

### Western blotting

The cells were harvested and incubated with cell lysis buffer overnight at −20°C. The protein concentrations of the extracts were determined using a BCA protein assay kit (Beyotime, Haimen, China). After electrophoresis, separated proteins were transferred to nitrocellulose membrane for 75 min at 110 V and blocked with 5% non-fat milk in TBS buffer for 1 h. After then, the membranes were washed with TBST buffer and incubated with primary antibodies overnight at 4°C and then secondary antibodies for 2 h at room temperature, followed by three washes in TBST buffer. The target proteins were detected on X-ray film using an enhanced chemiluminescence reagent. All the antibodies were obtained from Abcam and Cell Signaling Technology (Beverly, MA). β-actin was used to confirm the equal loading and transfer of proteins. The quantitative analysis of the target proteins was conducted by Quantity One. Changes in the levels of protein expression were shown as ratios of selected groups.

### Statistic analysis

Experiments were conducted at least three times and data was expressed as mean ± standard deviation (SD). Statistical analysis was carried out using SPSS statistical program version 13 (SPSS Inc., Chicago, IL). Difference between two groups was analyzed by two-tailed Student's t test and that between three or more groups was analyzed by one-way ANOVA multiple comparisons. Difference with *P* < 0.05 (*) or *P* < 0.01 (**) was considered statistically significant.

## Author Contributions

T.C. conceived and designed the experiments. W.C. performed the experiments. T.C. and W.Z. reviewed, analyzed and interpreted the data. W.C. and T.C. wrote the paper. All authors discussed the results and commented on the manuscript.

## Supplementary Material

Supplementary Information Supporting information

## Figures and Tables

**Table 1:** Cytotoxic effects of RuPOP on the selected human cancer and normal cell lines (t = 24 h, n = 3)

	IC_50_ (mean ± SD, μM)				
Complex	MDA-MB-231	MDA-MB-468	MCF-7	R-MCF-7^DOX^	HK-2
RuPOP	14.6 ± 3.1	78.0 ± 19.8	28.0 ± 4.9	55.5 ± 0.8	143.9 ± 10.2
