# Supplementary material for: Ruthenium Polypyridyl Complex Inhibits Growth and Metastasis of Breast Cancer Cells by Suppressing FAK signaling with Enhancement of TRAIL-induced Apoptosis
Source: Sci Rep. 2015 Mar 17;5:9157. doi: 10.1038/srep09157 (PMC4361883; doi:10.1038/srep09157)
Supplement: Supplementary Information — Supporting information [file srep09157-s1.doc]

**Supplementary Information**

**for**

**Ruthenium Polypyridyl Complex Inhibits Growth and Metastasis of Breast Cancer Cells by Suppressing FAK signaling with Enhancement of TRAIL-induced Apoptosis**

Wenqiang Cao, Wenjie Zheng, Tianfeng Chen*

Department of Chemistry, Jinan University, Guangzhou 510632, China

**Corresponding author:** Tianfeng Chen, tchentf@jnu.edu.cn.

**RESULTS**

**Figure S1. RuPOP and TRAIL synergistically inhibit growth of MDA-MB-231 cells but not in normal (HK-2) cell lines. (A)** RuPOP inhibits growth of MDA-MB-231 cells. Cells were treated with indicated concentrations of RuPOP for 48 h. **(B)** TRAIL inhibits growth of MDA-MB-231 cells. Cells were treated with indicated concentrations of TRAIL for 24 h. **(C)** Cytotoxic effects of RuPOP and/or TRAIL on human normal cell lines HK-2. Cells were pretreated with 20 µM RuPOP for 24 h and then expose to 40 ng/ml TRAIL for another 24 h. **(D)** RuPOP enhances the efficacy of TRAIL-induced MDA-MB-231 cells growth inhibition. Cells were pretreated with or without 2 µM RuPOP for 0, 12 or 24 h and then incubated in the presence or absence of 2 ng/ml TRAIL for another 24 h. Each value represents the mean ± SD of three independent experiments, *, *P* < 0.05; **, *P* < 0.01 versus the control.

**Figure S2**. Effects of LY294002 and U0126 on RuPOP-induced inhibition of MDA-MB-231 cells migration **(A)** and invasion **(B)**. All images shown are representative of three independent experiments with similar results.

**Figure S3**. Cytotoxic effects of RuPOP on MDA-MB-231 cells. (A) Cells were treated with 10, 20 and 40 µM of RuPOP for 6 and 12 h. Cell viability was examined by MTT assay. (B) Changes in the morphology of MDA-MB-231 cells under different treatments of RuPOP as examined by phasecontrast microscopy (magnification, 200×)

**Figure S4.** The full-length blots/gels of TfR.

**Figure S5.** The full-length blots/gels of total and phosphorylated FAK.

**Figure S6.** The full-length blots/gels of total and phosphorylated ERK and Akt.

**Figure S7.** The full-length blots/gels of MMP-2/-9, VEGF and TIMP-1.

**Figure S8.** The full-length blots/gels of uPA and uPAR.

**Figure S9.** The full-length blots/gels of caspase-3/-8/-9 and PARP.
